# Evaluating the Effect of Induction Chemotherapy on Bone Metabolizing Nutrients in Patients of Acute Lymphoblastic Leukemia

**DOI:** 10.7759/cureus.25403

**Published:** 2022-05-27

**Authors:** Aamir Khan, Junaid Zeb, Nazish Farooq, Nayab Farid, Rifaq Zeb, Muhammad Shoaib

**Affiliations:** 1 Hematology, Khyber Medical University, Peshawar, PAK; 2 Orthopedics, Khyber Medical College, Peshawar, PAK; 3 Pediatric Critical Care Medicine, Khyber Medical College, Peshawar, PAK; 4 Orthopedic Surgery, Hayatabad Medical Complex, Peshawar, PAK

**Keywords:** bone mass density, essential nutrients, chemotherapy, bone minerals, acute lymphoblastic leukemia (all)

## Abstract

Objective: To determine the levels of bone metabolizing nutrients (vitamin D, calcium, magnesium, potassium) in patients with acute lymphoblastic leukemia (ALL) before and after induction chemotherapy, and to correlate the effect of induction chemotherapy on their bone mass (BM).

Materials and methods: This quasi-experimental study was carried out at Hayatabad Medical Complex (HMC) and Khyber Medical University (KMU) in Peshawar, Pakistan, in one year. A total of 69 newly diagnosed patients with ALL were enrolled in the study. They were to begin the induction phase of chemotherapy at HMC oncology ward for about four weeks, following standard protocols. Data was collected using a predesigned questionnaire, and blood samples were obtained from all the patients by applying a non-probability consecutive sampling technique. The bone biomarkers levels were measured before therapy and after induction chemotherapy for comparison. Data analysis was performed using Statistical Package for the Social Sciences (SPSS) version 23 (IBM Corp., Armonk, NY, USA), and a p-value of <0.05 was considered significant.

Results: The mean age was 13 ± 5.23 years. Out of the 69 patients enrolled in the study, 36 (52%) were male and 33 (48%) were female. After the four-week induction chemotherapy, there was a significant reduction in bone contents levels. Vitamin D, calcium, magnesium and potassium levels were below the levels documented prior to the treatment with a p-value < 0.05. The bone mass remained unchanged after the four weeks of chemotherapy.

Conclusion: The induction phase of chemotherapy causes a significant reduction in the levels of bone bio contents and results in bone morbidities.

## Introduction

Acute lymphoblastic leukemia (ALL) is the most common and widespread hematological malignancy in the childhood years. In this type, the early lymphoid precursor cells proliferate and discontinue the differentiation process, as a result, symptoms of bone marrow failure appear including pancytopenia, fever, bleeding, infection, petechiae, rashes, central nervous system (CNS) symptoms, mediastinal masses and renal failure [1]. Worldwide a large number of patients are affected by ALL because of several types of mutations. Acute lymphoblastic leukemia is not a single disease but has specific phonotypic and genotypic variants that have their own diagnostic and therapeutic importance, and if not treated early can lead to significant mortality [2]. Globally, ALL is considered the third leading cause of death in children. Every year about 0.9 to 4.7 per 100,000 children are affected throughout the world [3]. In Pakistan, 32% prevalence of ALL was reported by one study, and 49.6% in Khyber Pakhtunkhwa (KPK), Pakistan [4,5]. The median age for ALL is 13 years and approximately 60% of cases are diagnosed up to the age of 25 which statistically accounts for about 75% to 80% of blood-related malignancy in children. The incidence rate is reportedly more between the age of two and three [6]. Over the past decades, therapeutic progress in ALL has been achieved. Once considered a globally lethal hematological malignancy, modern therapeutic techniques have gained outstanding outcomes. Treatment for pediatric ALL has improved in recent times and more than 80% of patients survive the disease after the early commencement of chemotherapy [7].

Chemotherapy is the primary treatment of ALL, consisting of four phases i.e. induction, consolidation, maintenance, and CNS prophylaxis. However, it is associated with multiple side effects. Skeletal morbidity is one of them, and high levels of skeletal morbidities such as musculoskeletal pains, bone fractures, and osteonecrosis are seen in a predominant number of ALL patients during chemotherapy [8]. Furthermore, dietary alteration, bone micronutrition, and deficiencies are also characteristics of childhood ALL and are linked with ALL chemotherapy [9]. In ALL patients, routine energy intake is insufficient and treatment with glucocorticoids in the induction phase of chemotherapy has an add-on risk for bone health. Gunes et al. observed an 85% decrease in bone minerals in ALL survival after chemotherapy [10]. Acute lymphoblastic leukemia and its chemotherapy affect the young population at an age when they gain peak bone mass density (BMD) and because of such reasons the bone mass is badly affected. Demidowicz et al. observed low levels of bone biomarkers in ALL patients after chemotherapy [11]. During induction chemotherapy, the different factors including chemotherapeutic agents, the steroids in it, low levels of supplements, child's age, and poor nutrition lead to bone structural changes and a decrease in its contents. This can lead to early osteoporosis in patients with ALL as well as other endocrine abnormalities that further contribute to the compromise in bone health [12]. Acute lymphoblastic leukemia is well-known leukemia that had significant mortality in the past. However, in the last few decades, the overall survival has improved, and risen to 90% if diagnosed and treated early [13]. The therapeutic outcomes are satisfactory because of the advanced chemotherapy protocols. Multiple studies conducted throughout the world to address bone health in leukemic patients have reported leukemic patients’ defective bone mineralization [14,15]. Limited literature is available locally in this regard. Therefore, this research work was done to determine the levels of some of the bone metabolizing nutrients (vitamin D, calcium, magnesium, potassium) in ALL patients before and after induction chemotherapy and to correlate the effect of induction chemotherapy on bone mass (BM) of ALL patients. This will aid in the diagnosis of bone morbidities during chemotherapy and will help oncologists and orthopedic experts to reduce its impact.

## Materials and methods

This was a quasi-experimental study carried out at Hayatabad Medical Complex (HMC) and Institute of Basic Medical Sciences (IBMS), Khyber Medical University (KMU) in Peshawar, Pakistan for a duration of one year (July 2019 to June 2020). Research began after receiving approval from the research ethics committee of KMU (approval no: DIR/KMU-EB/EE/000604). First, the identification and enrolment of ALL-affected patients were done. The patients were confirmed during the initial survey at HMC oncology OPD. By using a well-designed data questionnaire, patients were interviewed to record the history of incidence and status of the disease. After enrolment, the purpose of the study was explained, and written informed consent was obtained. Also, patients' data regarding sociodemographic and anthropometric characteristics were recorded. Non-probability consecutive sampling method was adopted for the sample section. For investigating the bone biomarkers, a total of 69 ALL-affected individuals were recruited under inclusion and exclusion criteria with the following assumptions: anticipated reduction of 84% in bone mineral content after chemotherapy in patients with ALL (previous study), a confidence level of 95%, absolute precision at 9%. According to the criteria, those who were newly diagnosed with ALL and aged between two to 25 years of both genders were included. Patients who were taking mineral supplements, who are already on ALL chemotherapy, and aged above 25 years were excluded.

Baseline (5cc) blood samples were obtained from the antecubital vein of the selected patients using aseptic techniques. These patients were then followed up after four weeks of induction chemotherapy for 28 days. After the completion of chemotherapy, 5cc of blood was once again collected using aseptic techniques for comparison of bone metabolizing nutrients. The blood samples (both before and after induction chemotherapy) were transported from the collection spot at room temperature in thermophile boxes, serum was extracted through centrifugation and was stored at 4ºC to 8ºC for further analysis. Four different biochemical parameters were analyzed by using different techniques. The magnesium and potassium levels were determined with the Cobas c-501 electrolyte analyzer (Roche Diagnostics, Bassel, Switzerland) which works on the principle of ion-selective electrodes (ISE). The calcium level was determined by using the Cobas c-111 electrolyte analyzer. The enzyme-linked immunosorbent assay (ELISA) method was used to determine the level of vitamin D. The Mi body composition scale (Xiaomi, Beijing, China) was used to measure the bone mass in ALL-affected patients before and after induction chemotherapy. Statistical analysis was done using Statistical Package for Social Sciences (SPSS) version 23.0 (IBM Corp., Armonk, NY). Continuous variables were reported as mean and standard deviation and categorical variables as numbers (percentage). Paired sample T-tests were applied and the level of significance was set at a p-value less than 0.05 (p<0.05).

## Results

The mean age was 13 ± 5.23 years. Thirty-six (52%) patients were male and 33 (48%) were female. The baseline hematological parameters and bone biomarkers, namely hemoglobin (Hb), white blood cells (WBCs), platelets, vitamin D, calcium, potassium, magnesium, and bone mass, were measured in all the patients and are summarized in Table 1.

**Table 1:** Baseline hematological parameters of the study population (n=69) ALT: Alanine triphosphate, ALP: Alkaline phosphatase

Parameters	Minimum	Maximum	Mean ± SD
WBCs	1.30 x10^9^/L	88.0 x10^9^/L	9.41±12.7
Platelets	1.50 x10^9^/L	5.20 x10^9^/L	3.22±0.79
Bilirubin (mg/dl)	0.1	1.10	0.54±0.18
ALT (IU/L)	7.00	186.00	40.8±37.2
ALP (IU/L)	55.0	265.00	151.5±51.9
Hemoglobin (g/dl)	2.40	13.30	8.67 ±2.26
Magnesium	1.20	2.70	2.01 ±0.35
Calcium	7.89	10.10	9.27 ±0.49
Potassium	3.80	5.80	4.92±0.47
Vitamin D	5.38	42.10	22.2 ±9.51
Bone mass	1.40	2.60	1.96 ±0.38

A comparison between pre- and post-treatment hematological parameters is shown in Table 2. Our results revealed that after treatment, there was a significant decrease observed in the levels of vitamin D, calcium, magnesium, and potassium (p<0.05). However, the correlation and T-test for bone mass cannot be computed because of the null difference in the standard error.

**Table 2:** Comparison of pre-treatment and post-treatment micronutrients (n=69) NS: Not significant

Variables	Pre-treatment Measurements	Post-treatment Measurements	Difference	Pre-treatment & Post-treatment Minimum	Difference		Pre-treatment & Post-treatment Maximum	Differnce	p-value
Magnesium (g/dl)	2.00±0.4	1.66±0.6	0.34±-0.3	0.00-1.20	-1.2	2.60-2.7		-0.1	<0.016
Calcium (g/dl)	9.27±0.5	8.31±2.9	0.96±-2.4	0.00-7.89	-7.89	10.2-10.1		0.1	0.008
Potassium (meq/l)	4.92±0.5	4.23±1.5	0.68±-1.0	0.00-3.80	-3.80	5.60-5.80		-0.2	<0.047
Vitamin D (ng/ml)	22.2±9.5	19.3±10.8	2.89±-1.3	0.00-538	-538	42.1-42.1		0.0	<0.06
Bone mass (kg)	1.96±0.4	1.96 ±0.3	0.00±0.0	1.40-1.40	0.0	2.60-2.6		0.0	NS

To determine the difference in the levels of biomarkers between male and female genders, the p-value was calculated by using the paired sample T-test. There was no significant difference found in calcium levels in female patients pre- and post-induction chemotherapy. However, the rest of the parameters were found to be low after induction chemotherapy in both male and female patients (p<0.05). The correlation and T-test for bone mass cannot be computed because of the null difference in the standard error. The results are summarized in Table 3.

**Table 3:** Pre-treatment and Post-treatment side effects significance in male and female patients NS: Not significant

	In male patients			In female patients		
Variables	Pre-treatment Measurements	Post-treatment Measurements	p-value	Post-treatment Measurements	Post-treatment Measurements	p-value
Vitamin D (ng/ml)	22.1±9.3	17.7±11.8	0.002	22.3 ±9.9	20.9±9.3	0.015
Calcium (g/dl)	9.25±0.5	7.46±3.7	0.008	9.29±0.5	9.24±0.5	0.528
Magnesium (g/dl)	1.96±0.3	1.46±0.8	0.001	2.05±0.3	1.88±0.4	0.012
Potassium (meq/l)	4.93±0.4	3.77±1.9	0.001	4.91±0.5	4.75±0.5	0.018
Bone mass (kg)	1.96±0.4	1.96 ±0.4	NS	1.96±0.4	1.96±0.4	NS

## Discussion

Acute lymphoblastic leukemia is one of the most common hematological malignancies found in early childhood. The prognosis of this disease is above 90% if early treatment with anti-leukemic agents is introduced on time. Acute lymphoblastic leukemia chemotherapy is associated with different side effects including osteopathy, especially in early childhood as compared to adults. The exact mechanism of bone damage in ALL is not fully understood but the infiltration of leukemic cells into the microenvironments of bone cells, and a decrease in the bone mass and bone minerals due to chemotherapy are proposed possible causes [16]. The purpose of this study was to evaluate the pre- and post-treatment induction effects of chemotherapy on bone metabolizing nutrients in patients affected by ALL and to identify the effect of ALL and chemotherapy on bone health.

In our study, it was found that bone metabolizing nutrients are profoundly affected by the four-week induction phase of chemotherapy. Bone-related morbidities are associated with alterations in calcium, vitamin D, magnesium, and potassium levels. In our study, the level of calcium was found to be low in all patients (p<0.05) after induction chemotherapy. The results are similar to the findings of another study by van der Sluis et al. [17]. Another study with similar findings states that the bone mineral level especially that of calcium decreased when ALL-affected patients were treated with cytotoxic therapy. The abnormal level of calcium in ALL affects the bones' health. One other study demonstrated that when ALL-affected children are exposed to chemotherapy, particularly steroids, it leads to osteopenia.

Similarly in our study, a significant reduction in vitamin D levels is also observed (p<0.05). Our findings are in correspondence with the results of Simmons et al. who described a 15% reduction in vitamin D levels after induction chemotherapy [18]. A case-control study by Reisi et al. observed low levels of vitamin D in 27% of ALL survivors [19]. Likewise, another study has documented the low frequency of vitamin D in children aged >4 years during methotrexate therapy causing bone diseases [20].

Our study also revealed a significant reduction in the level of magnesium after induction chemotherapy. Previous studies conducted in ALL-affected children have also reported low magnesium levels in the body after chemotherapy completion. The study conducted by Sahin et al. also observed a low level of magnesium in association with chemotherapy in patients with ALL [21]. Magnesium supplementation is needed for ALL-affected individuals to prevent hypomagnesemia. Furthermore, potassium, which is another essential bone metabolizing content, was at decreased levels in our patients after induction chemotherapy and caused bone weakness and muscle pain. Our findings are parallel with the results of other studies that report persistent hypokalemia in ALL patients during induction chemotherapy [22].

There is no significant change observed in the bone mass of our study which is in contradiction with the results of previous studies in which altered bone mass has been reported [23]. The difference in results may be due to the different chemotherapy regimens used in our setup. Chemotherapy is used successfully for the treatment of ALL. However, several research studies on bone metabolizing nutrients in ALL-affected children in different regions of the world show that chemotherapy has adverse effects on bone health, and bone metabolizing nutrients levels decrease as the treatment progresses.

Our study has some limitations as it was a single-center study and not all the parameters of bone metabolism were measured. There is room for more research work on bone health in leukemic patients.

## Conclusions

Our study's findings and results concluded that bone health is significantly affected during the induction phase of the chemotherapy in ALL patients and can lead to severe bone-related morbidities. The cause is a significant reduction in bone metabolizing nutrients of vitamin D, calcium, magnesium, and potassium post the induction chemotherapy. Therefore, it is suggested that early recognition of these morbid conditions is necessary and preventive measures in the form of supplements must be provided to these patients at the time of diagnosis or at the start of induction chemotherapy to minimize these osteopathies.
